# Dielectric Relaxation, Local Structure and Lattice Dynamics in Mn-Doped Potassium Tantalate Ceramics

**DOI:** 10.3390/ma14164632

**Published:** 2021-08-17

**Authors:** Alexander Tkach, Abilio Almeida, Igor Levin, Joseph C. Woicik, Paula M. Vilarinho

**Affiliations:** 1 Department of Materials and Ceramic Engineering, CICECO–Aveiro Institute of Materials, University of Aveiro, 3810-193 Aveiro, Portugal; paula.vilarinho@ua.pt; 2 Department of Physics of Science Faculty, IFIMUP, University of Porto, 4169-007 Porto, Portugal; amalmeid@fc.up.pt; 3 Materials Measurement Science Division, National Institute of Standards and Technology, Gaithersburg, MD 20899, USA; igor.levin@nist.gov (I.L.); woicik@bnl.gov (J.C.W.)

**Keywords:** electroceramics, perovskites, polar dielectrics, X-ray absorption, Raman spectroscopy

## Abstract

Alkaline niobate and tantalate perovskites have attracted attention as polar dielectrics for electronics and telecommunications. Here, we studied the polar behaviour, lattice dynamics, and local structure in conventionally processed K_0.985_Mn_0.015_TaO_3±δ_ ceramics using a combination of variable-temperature dielectric and Raman spectroscopies, and X-ray absorption fine structure (XAFS) measurements, respectively. Mn doping induces a low-frequency dielectric relaxation in KTaO_3_ (KT), which follows the Arrhenius law with an activation energy *U* ≈ 105 meV and the characteristic relaxation time *τ*_0_ ≈ 4.6 × 10^−14^ s. Our XAFS results support preferential Mn occupancy of the cuboctahedral sites as Mn^2+^, with these cations strongly off-centred in the oversized oxygen cages. Such disordered Mn displacements generate electric dipoles, which are proposed as the source of the observed dielectric relaxation. We show that in Mn-doped ceramics, the low-frequency polar TO1 mode softens on cooling and, at low temperatures, exhibits a higher frequency than in undoped KT. This mode displays no detectable splitting, which contrasts with Li-doped KT that also contains off-centred Li^+^ species on the cuboctahedral sites. Therefore, we conclude that the coupling between the Mn displacements and the lattice is weaker than in the Li case, and Mn-doped KT therefore exhibits a dielectric relaxation but no ferroelectric transition.

## 1. Introduction

Perovskite-structured compounds SrTiO_3_ (ST), KTaO_3_ (KT) and CaTiO_3_ (CT) stand out as incipient ferroelectrics, with their dielectric permittivity increasing continuously on cooling due to the polar-mode softening but without a ferroelectric phase transition [1]. Incipient ferroelectrics exhibit strong dependence of the real part of the dielectric permittivity, ε′, on electric field and small values of the dissipation factor, tanδ, which makes them attractive for applications in tunable electronic components [2,3]. Among such ABO_3_ systems, however, only KT retains an ideal cubic perovskite structure with *Pm-3m* symmetry down to 0 K [4].

Mn is a common dopant in transition metal oxides. The mechanisms for its accommodation in host lattices of perovskite incipient ferroelectrics has been studied most extensively for SrTiO_3_ [5]. Depending on substitution formulations (i.e., for Sr or Ti) and annealing conditions (temperature, partial oxygen pressure), Mn has been shown to adopt mainly Mn^4+^ and Mn^2+^ oxidation states. While ST systems containing exclusively Mn^4+^ could be obtained, Mn^2+^ has always coexisted with Mn^4+^ or Mn^3+^. The site preference for Mn varies with its oxidation state and the resulting effective ionic radius. The smaller Mn^4+^ species always occupy octahedral B sites, whereas the significantly larger Mn^2+^ cations prefer cuboctahedral A sites [5]. A recent study of Mn doping in CT revealed dissimilar behaviour compared to that in Mn-doped ST. In CT, Mn is present in the mixed Mn^4+^/Mn^2+^ state regardless of its substitution for Ca or Ti in the formula, with Mn^4+^ and Mn^2+^ cations located in the octahedral and cuboctahedral sites, respectively [6]. In both ST- and CT-based systems, Mn^2+^ species are strongly off-centred in the oversized cuboctahedral cages, generating electric dipoles. The presence of such positional disorder for Mn^2+^ has been invoked as an explanation for the dielectric relaxation and multi-glass (i.e., dipolar plus spin glass) behaviour observed in Mn-doped ST [7]. The multi-glass state has been associated with polar displacements of Mn^2+^ in the highly polarizable incipient ferroelectric lattice, thereby initiating a transition of the Mn^2+^ magnetic moments into a spin glass.

KT-based compounds have mainly been investigated as single crystals [8,9]. Although ceramics are assumed to possess higher and more controllable dopant contents, being also cheaper to fabricate than single crystals, the scarcity of works on ceramics can be attributed to the relatively low melting temperature of KT, which, combined with the high volatility of potassium, makes it challenging to synthesize dense monophasic ceramics with well-controlled chemical compositions [10]. Among KT-based compounds, K_1−*x*_Li*_x_*TaO_3_ solid solutions have been studied intensively, wherein displacements of small Li^+^ ions on K sites generate strong local dipole moments that couple electrostatically to the KT’s polar soft mode [8,9]. As a result, both dielectric relaxations and a ferroelectric phase transition were reported for the K_1−*x*_Li*_x_*TaO_3_ system [8,9,11,12,13]. Similar to Li-doped KT and Mn-doped ST, Mn-doped (0.01% to 0.3%) KT single crystals [14,15,16] exhibit a dielectric relaxation which has also been attributed to a positional disorder of Mn^2+^ on K sites [15]. 

Mn-doped KT ceramics [17,18,19] feature a relaxation at low frequencies, while in the microwave range, the relaxational effects are manifested in tanδ, but not in ε′ [19,20,21]. The K_0.97_Mn_0.03_TaO_3±δ_ ceramics [17,18] display both dielectric and magnetic anomalies, which resemble the “multi-glass” behaviour observed in Mn-doped ST [7,22]. However, the interpretation of the magnetic response in K_0.97_Mn_0.03_TaO_3±δ_ is complicated because of a contribution from the second phase, MnO*_y_*. Indeed, MnO*_y_* has been detected in elemental maps of K_0.97_Mn_0.03_TaO_3__±δ_ ceramics, whereas K_0.985_Mn_0.015_TaO_3__±δ_ and KTaO_3_ have displayed homogeneous elemental distributions devoid of chemical segregation while featuring distinct lattice parameters [23]. A study of the magnetic response in K_1-2*x*_Mn*_x_*TaO_3_ loose powders, which have been formulated with the intentional K deficiency to provide charge compensation for the aliovalent Mn^2+^ substitution, claimed the formation of Mn_3_O_4_ for *x* ≥ 0.01 [24]. However, the same group reported contradictory data on the Mn solubility limit for this formulation by demonstrating a linear dependence of the lattice parameter on Mn content up to *x* = 0.03 [20]. For *x* = 0.04, a Ta-rich tungsten bronze structure was detected as the main extra phase [20]. In addition to this apparent controversy regarding the Mn solubility limit in the loose powders [20,24], K_1__−__2*x*_Mn*_x_*TaO_3_ ceramics exhibited a non-monotonic trend for the dielectric relaxation, with the relaxation strength being the strongest for *x* = 0.01, significantly diminished for *x* = 0.02 and 0.03, and partially restored for *x* = 0.05 [19,24]. Such an inconsistent behaviour of the relaxation suggests that for *x* ≥ 0.02, K_1-2*x*_Mn*_x_*TaO_3_ ceramics contain other phases. Overall, the available data indicate that for the K_1-2*x*_Mn*_x_*TaO_3_ nominal stoichiometry, a controlled Mn substitution is difficult to achieve.

Thus far, little research has been undertaken into the local structure and lattice dynamics in Mn-doped KT ceramics or single crystals, especially as a function of temperature. Here, we combined variable-temperature dielectric measurements over a broad frequency range, room-temperature X-ray absorption fine structure (XAFS) measurements, and variable-temperature Raman spectroscopy to determine the site occupancy and coordination environments for the Mn dopant species in KT ceramics that have been confirmed as monophasic [23].

## 2. Materials and Methods

For this study, we selected ceramics with the nominal K_0.985_Mn_0.015_TaO_3__±δ_ composition prepared using conventional solid-state synthesis, which has been demonstrated to yield a single perovskite phase [23]. The K_2_CO_3_ (Merck KGaA, Darmstadt, Germany, purity 99+%), Mn(NO_3_)_2_ (Merck KGaA, Darmstadt, Germany, purity 98.5+%) and Ta_2_O_5_ (Sigma-Aldrich, Saint Louis, MO, USA, purity 99+%) reagents (Certain commercial products or company names are identified here to describe our study adequately, not intending to imply recommendation or endorsement by National Institute of Standards and Technology (NIST), nor is it intended to imply that the products or names identified are necessarily the best available for the purpose.) were dried to remove moisture, mixed, and milled in alcohol for 5 h in a planetary mill using Teflon containers and zirconia spherical grinding media. The loss of potassium, which is expected to occur during sintering, was compensated by adding 5 wt.% excess of potassium to the initial mixture prior to the milling [10]. The resulting powders were dried and calcined at 875 °C for 8 h. The calcined powders were milled again for 5 h to reduce the particle size below 5 μm, and uniaxially pressed (100 MPa) into pellets, 10 mm in diameter. The pellets, covered with powder of the same composition to mitigate the loss of potassium, were sintered in closed alumina crucibles in air at 1350 °C for 1 h with a heating and cooling rate of 5 °C/min, similarly to the conditions used previously for preparing undoped and Li-doped KT ceramics [11]. After sintering, the pellets exhibited a density of ≈88% and an average grain size of ≈1.8 μm.

For dielectric measurements, gold electrodes were sputtered on both sides of the polished ceramic pellets. Complex dielectric permittivity, including its real *ε*′ and imaginary *ε*″ parts, were measured in the frequency range from 10^2^ Hz to 10^8^ Hz between room temperature and 10 K using a precision LCR-meter (HP 4284A, Hewlett Packard, Palo Alto, USA) and a RF Impedance Analyzer (HP 4191A, Hewlett Packard, Palo Alto, CA, USA). The dissipation factor was calculated as tanδ = *ε*″*/ε*′. Unpolarised Raman spectra were recorded in the spectral range from 10 cm^−1^ to 1000 cm^−1^ in a pseudo-backscattering geometry using a spectrometer (Jobin-Yvon T64000, Horiba, Kyoto, Japan) equipped with a charge-coupled device and a photon-counting detector. The spectral slit width was about 1.5 cm^−1^, while an excitation was performed using a Spectra Physics argon laser operating at a wavelength λ = 514.5 nm. The spectra were registered at a series of temperatures between 10 K to 290K, after a waiting time of 15 min at each temperature. During the dielectric and Raman-spectroscopy measurements, temperature (*T*) control was achieved using a He closed-cycle cryogenic system (Displex APD-Cryostat HC-2, Allentown, PA, USA), equipped with silicon diode temperature sensors and a digital temperature controller Scientific Instruments Model 9650. XAFS measurements for the Mn *K*-edge (6539 eV) were conducted at ambient temperature at the NIST X23A2 beamline of the National Synchrotron Light Source (NSLS, Brookhaven National Laboratory) on finely ground (mortar and pestle) powders dispersed on a double-sided scotch tape. The double-crystal monochromator was operated with a pair of Si (311) crystals. About 50 spectra were collected in the fluorescence mode using a four-element Si-drift detector. The detector count rate was adjusted to minimize the dead-time effects [25]. Portions of X-ray absorption spectra containing near-edge (XANES) and extended (EXAFS) fine structure were recorded in the same run using smaller energy steps for XANES and longer counting times for EXAFS. Transmission data from a Mn foil positioned downstream of the sample was recorded simultaneously with each scan for energy calibration. Mn spectra for the reference samples of SrMn^4+^O_3_, YMn^3+^O_3_, and Mn^2+^TiO_3_ have been measured previously at the same beamline. These measurements were performed on powder samples in transmission. The XAFS data were processed and analysed using Athena and Artemis software, respectively [26]. Scattering phases and amplitudes were calculated using FEFF8 [27].

## 3. Results and Discussion

**Figure 1:**
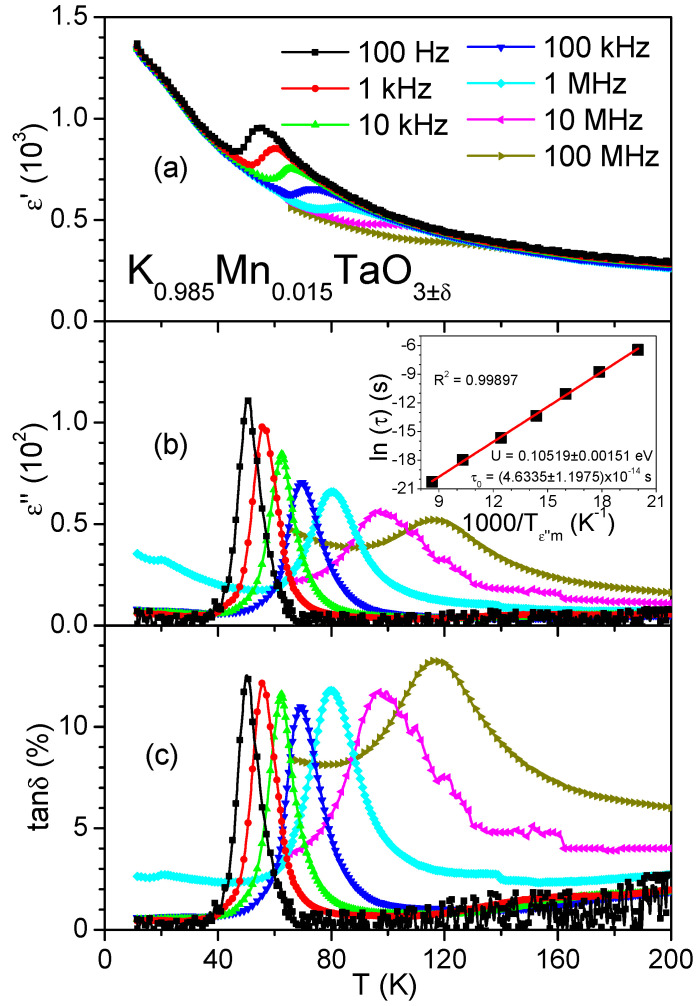
Real *ε*′ (**a**) and imaginary *ε*″ (**b**) parts of the dielectric permittivity and dissipation factor tanδ (**c**) of K_0.985_Mn_0.015_TaO_3±δ_ ceramics as a function of temperature *T* in the 100 Hz–100 MHz frequency range. Inset shows the Arrhenius plot ln(*τ*) versus 1000*/T_ε_*_″m_ for the dielectric relaxation of K_0.985_Mn_0.015_TaO_3±δ_ ceramics with fits to the Arrhenius law (solid line) and the fit parameters.

As reported previously, undoped KT ceramics upon cooling display a continuous increase of the dielectric permittivity to *ε*′ ≈ 4000 without frequency dispersion [10,11]. In contrast, for the Mn-doped KT ceramics, the maximum attainable value of *ε*′ is only ≈1400 (see Figure 1a), and the *ε*′(*T*) dependence reveals a diffuse peak with a frequency-dependent amplitude and position, suggesting a dielectric relaxation. This relaxation is also manifested by the frequency-dependent peaks in both *ε*″(*T*) (see Figure 1b) and *tanδ*(*T*) (see Figure 1c), with the peak-maximum temperature varying from about 50 K at 100 Hz to about 116 K at 100 MHz. Our results reconcile the observation of a permittivity peak for frequencies up to 10 MHz by Shvartsman et al. [18] and the absence of such a peak at microwave frequencies between 2.5 GHz and 3.5 GHz by Axelsson et al. [20,21]. Indeed, the peak in *ε*′(*T*) becomes invisible above 10 MHz, but the corresponding peaks in *ε*″(*T*) and *tanδ*(*T*) are still observed even above this frequency. For the K_0.97_Mn_0.03_TaO_3±δ_ ceramics studied in [18], the dielectric relaxation occurred at approximately the same temperatures as observed here for K_0.985_Mn_0.015_TaO_3__±δ_, but it appears to be stronger, which is consistent with a larger number of independent dipoles created for the higher Mn content.

We clarified the origins of electric dipoles responsible for the relaxation by using the Debye approximation to analyse relaxational dynamics from the behaviour of the maximum of the *ε*″(*T*) peak as a function of temperature and frequency. In this approach, a set of independent dipoles is characterized by a unique relaxation time (*τ*), which is equal to the inverse of the angular relaxation frequency (*ω* = 2π*f*). The relaxation is described using the Arrhenius law:

*τ* = *τ*_0_exp(*U*/k_B_*T*) (1)

where *τ*_0_ is the relaxation time at infinite temperature. Here, *U* is the activation energy of the dipolar process, k_B_ is the Boltzmann constant, and *T* is the absolute temperature. The dynamics of the diffuse peak in the frequency range from 10^2^ Hz to 10^8^ Hz can be examined using a plot (inset in Figure 1b) of ln(*τ*) vs. 1000/*T_ε_*_″m_, where *T_ε_*_″m_ is the temperature that corresponds to the maximum of the *ε*″(*T*) peak at the angular frequency *w = 2πf* = *τ*^−1^. From this plot, we find *U* = 105 meV and *τ*_0_ = 4.6×10^−14^ s, which are close to the corresponding values determined for Mn-doped (0.01% to 0.3%) KT single crystals using dielectric-spectroscopy and electron-spin-resonance (ESR) measurements [14,15], as shown in Table 1. Our values of *U* and *τ*_0_ are also close to those reported for Mn-doped (0.5% to 5%) KT ceramics [18,19] (Table 1), with the caveat that Mn content in some of these compositions exceeded the solubility limit.

Since a prior microscopic characterization of the present ceramics confirmed a homogenous distribution of Mn within the KT lattice [23], the observed dielectric relaxation should have intrinsic origins. For (001)-oriented Mn-doped KTaO_3_ single crystals, studied by dielectric spectroscopy and ESR techniques, the relaxation with the activation energy of 104–110 meV has been proven to originate from polar displacements of Mn^2+^ cations occupying the K sites [15]. Although ceramics differ from single crystals, possessing grain boundaries and pores but having no preferred crystallographic orientation, similar activation energies in our ceramics and the previously studied Mn-doped KT single crystals [15] indicate that the dielectric relaxation observed here can be related to the same disordered-ion-displacement mechanism [8,15].

**Figure 2:**
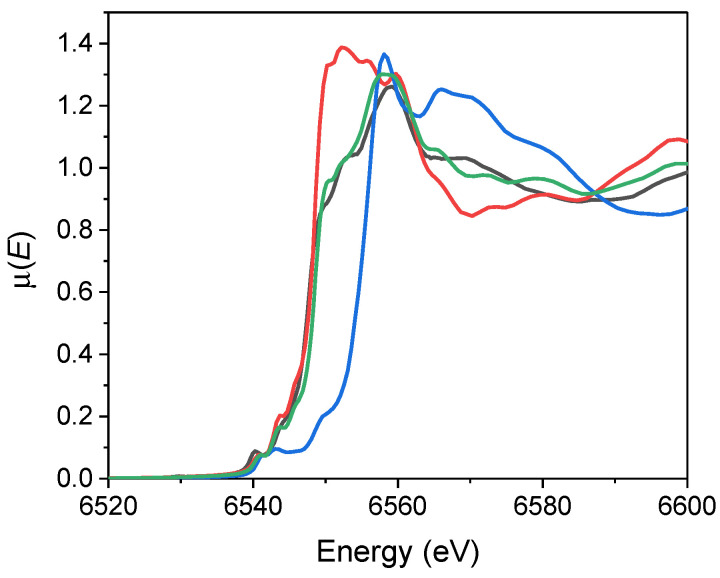
Mn *K*-edge X-ray absorption near-edge structure (XANES) for K_0.985_Mn_0.015_TaO_3±δ_ (black), MnTiO_3_ (red), SrMnO_3_ (blue). A fit of the K_0.985_Mn_0.015_TaO_3±δ_ XANES using a linear combination of the spectra for MnTiO_3_ and SrMnO_3_ is shown in green.

**Figure 3:**
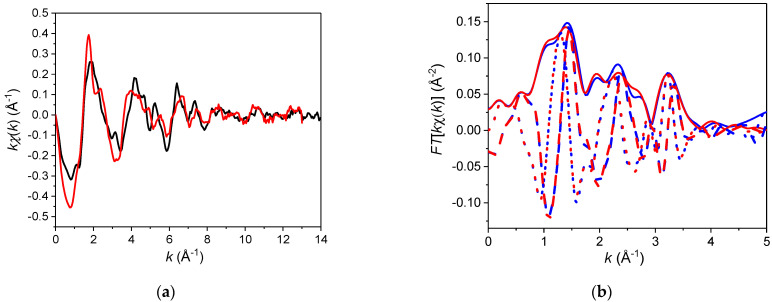
(**a**) Experimental *k*-weighted (where *k* is the wavevector) EXAFS signal for the present K_0.985_Mn_0.015_TaO_3±δ_ (red) and previously analysed [29] Sr_0.98_Mn_0.02_TiO_3_ (black) ceramics. The latter composition was shown to contain a 0.6:0.4 mixture of Mn^2+^ and Mn^4+^ residing on the A- and B-sites, respectively. Note the overall similarity between the two signals, with differences accounted for by different backscattering species in the second- and higher-order coordination shells. (**b**) Experimental (blue) and fitted (red) Fourier transform (FT) of the EXAFS signal for K_0.985_Mn_0.015_TaO_3±δ_. The magnitude of the FT is shown using solid lines, whereas the real and imaginary parts of the FT are indicated using dashed and dotted lines, respectively. The *k* range used in the FT was 2.68 Å^−1^ to 10.98 Å^−1^. The *R* factor describing the quality of the fit is 0.0095.

Our XAFS results support this inference, providing evidence for the presence of Mn on the cuboctahedral sites in KT as strongly off-centred Mn^2+^ species. Figure 2 compares XANES for Mn in K_0.985_Mn_0.015_TaO_3±δ_, Mn^2+^TiO_3_, and SrMn^4+^O_3_. Clearly, the doped KT ceramics contain a significant fraction of Mn^2+^. A linear combination fit using MnTiO_3_ and SrMnO_3_ as references provides a reasonably good match to the spectrum for the doped KT and therefore suggests that the latter contains a mixture of Mn^2+^ and Mn^4+^ cations with the 2:1 ratio. Both XANES and EXAFS (Figure 3a) for K_0.985_Mn_0.015_TaO_3±δ_ are similar to those for the previously studied Sr_0.98_Mn_0.02_TiO_3_ ceramics, which have been shown to contain a mixture of Mn^2+^ and Mn^4+^ residing on the A- and B-sites, respectively. 

Given the mixed oxidation state of Mn suggested by the XANES, we tested two models for the coordination of Mn while fitting the EXAFS data (Figure 3b). Model 1 assumed the presence of Mn on both octahedral (as Mn^4+^) and cuboctahedral (Mn^2+^) sites. In contrast, Model 2 considered a mixture of two different octahedral sites populated by the smaller Mn^4+^ (ionic radius 0.53 Å) and larger Mn^2+^ (0.83 Å [28]) species, respectively. The Mn EXAFS data are insufficient to support reliable refinements of all the distances and their associated Debye-Waller (D-W) factors for the two coordination environments as independent variables [29]. Therefore, we adopted a previously used strategy of fixing most of the structural parameters for the rigid Mn^4+^ octahedral coordination at their well-characterized values for Mn^4+^ in doped SrTiO_3_ (see the footnote to Table 2 for more details). The parameters describing the coordination of Mn^2+^ in Models 1 and 2 were treated as variables; a fraction of the Mn^2+^ species was refined as well. For the K-site coordination, only single-scattering paths of the photoelectron were included in the fit since in this case the contributions of multiple-scattering events are negligible. For the octahedrally coordinated Mn, we considered both single and multiple scattering paths.

Model 2 provided a poor fit to the data, especially in the *r*-range with strong multiple-scattering contributions, which are characteristic of the octahedral coordination in perovskites. Additionally, some of the refined structural variables acquired unphysical values. Therefore, we discarded this model. In contrast, Model 1 reproduced the data satisfactorily with sound values for the parameters (Table 2), which overall were consistent with the features of Mn^2+^ in the cuboctahedral coordination reported previously for Mn-doped ST and CT. The refined fraction of the cuboctahedral Mn was 0.73 ± 0.04, consistent with the estimate for the Mn^2+^ species from the linear combination fit of the XANES. As observed in other perovskite compounds, the relatively small Mn^2+^ cations (ionic radius ≈1.27 Å in the cuboctahedral coordination [30]) are significantly off-centred within the K-site oxygen cages, forming several short Mn-O bonds. The refined values of the D-W factors indicate a significant spread even for such short distances. Additionally, the Mn^2+^-Ta distance appears to be shorter than expected from the average KT structure, suggesting a relaxation of the Ta cations around the K-site Mn. We feel, however, that the present EXAFS data are too limited for a more detailed understanding of structural relaxations induced by Mn.

Interestingly, we obtained similar XANES and EXAFS for KTa_0.985_Mn_0.015_O_3__±δ_ ceramics synthesized under the conditions identical to those described in the experimental section. This similarity points to an amphoteric behaviour of Mn dopants in KT, with the preferred oxidation states and site occupancies being independent of Mn substitution for K or Ta in the chemical formula. This behaviour resembles that recently reported for Mn in CT. In the latter system, it was attributed to octahedral rotations which stabilize Mn^2+^ on the cuboctahedral sites [6]. In KT, the preferential occupancy of Mn^2+^ on the K-site can be promoted by the difficult-to-avoid K deficiency caused by the volatility of these species.

**Figure 4:**
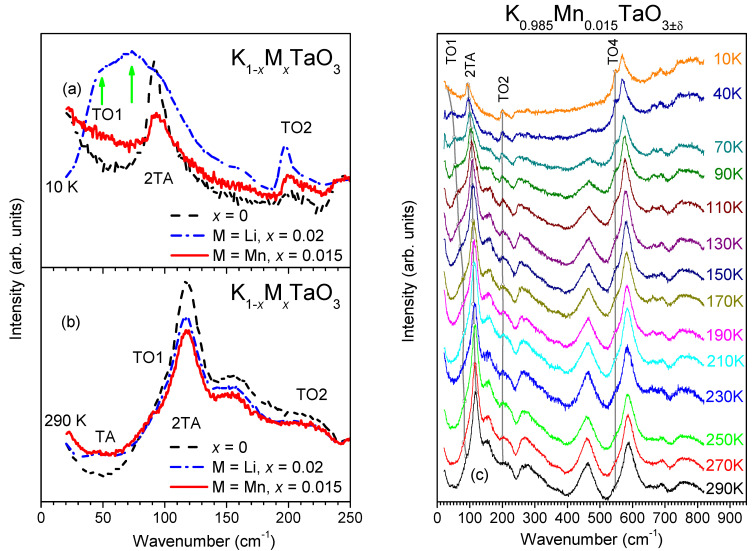
(**a**,**b**) Lower-wavenumber portions of Raman spectra for K_0.985_Mn_0.015_TaO_3±δ_ ceramics (solid lines) at 10 K (**a**) and 290 K (**b**) compared to those of ceramic KTaO_3_ (dash lines) and K_0.98_Li_0.02_TaO_3_ (dash dot lines) recorded at the same temperatures. (**c**) Raman spectra of the K_0.985_Mn_0.015_TaO_3±δ_ ceramics over a broad wavenumber range recoded between 10 K and 290 K (**c**).

In the case of Li doping, the dielectric relaxations induced by off-centre displacements of Li^+^ ions on K sites are accompanied by a ferroelectric phase transition [8,9,13]. This transition is reflected in the split TO1 mode in low-temperature Raman spectra for the Li content greater than 1.4% [31], as shown by arrows in Figure 4a for K_0.98_Li_0.02_TaO_3_ ceramics [12]. In contrast, for the present K_0.985_Mn_0.015_TaO_3__±δ_ ceramics, the spectra display no pronounced TO1 mode, as also observed for undoped KT [12]. At room temperature, all three compositions yield similar spectra (Figure 4b). 

Figure 4c illustrates the evolution of spectra for the Mn-doped KT ceramics as a function of temperature. First-order Raman scattering in both undoped and Mn-doped KT is forbidden by their cubic *Pm-3m* symmetry so that most of the features seen in Figure 4 arise from second-order Raman processes. However, the broad features around 50 (labelled as TA) and 110 cm^−1^ (2TA) that are visible in the room-temperature spectra but gradually disappear on cooling are related to the two-phonon scattering linked to a peak in the density of states of the transverse acoustic (TA) branch at the Brillouin zone boundary [31,32]. Several Raman features emerge on cooling, including those from the infrared-active optical modes around 547 cm^−1^ (TO4) and 200 cm^−1^ (TO2), as well as a broad, low-frequency shoulder assigned to the TO1 mode [32]. The appearance of these optical modes in ceramics has been attributed to frozen electric dipoles at grain boundaries, resulting in the local loss of inversion symmetry, thus breaking the selection rules for Raman and infrared activities [33].

**Figure 5:**
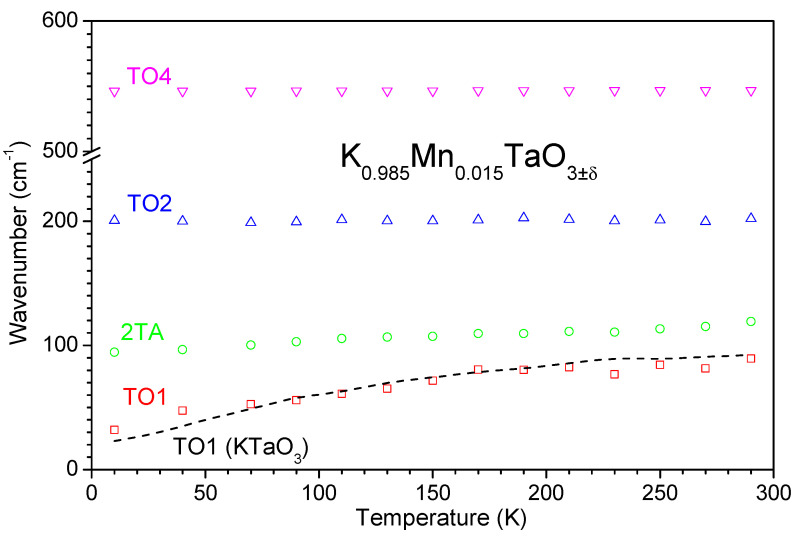
Variation of the TO1 (squares), 2TA (circles), TO2 (up triangles), and TO4 (down triangles) Raman modes in the K_0.985_Mn_0.015_TaO_3±δ_ ceramics with temperature. The behaviour of the TO1 mode for undoped KT ceramics (dashed line) is shown for comparison.

We fitted the first-order features in the background-subtracted Raman spectra using a model of independent damped oscillators [12]. According to this model, the Raman intensity *I*(*ω*,*T*) is described as

(2) $$I\left(\omega ,T\right)=\left(1+n\left(\omega ,T\right)\right)\overset{N}{\underset{j=1}{\sum }}\left(A_{oj}\frac{\omega \Omega_{oj}^{2}\Gamma_{oj}}{{\left(\Omega_{oj}^{2}-\omega^{2}\right)}^{2}+\omega^{2}\Gamma_{oj}^{2}}\right)$$

where *ω* is the frequency, *T* is the temperature, *n*(*ω*,*T*) is the Bose-Einstein factor, and *A_oj_*, *Ω_oj_*, and *Γ_oj_* are the strength, the wavenumber, and the damping coefficient for the *j*-th oscillator, respectively. The fitted frequencies of the TO1, TO2, TO4, and 2TA modes for the doped ceramics are plotted in Figure 5 as a function of temperature. While frequencies of the TO4 and TO2 modes remain approximately constant, the TO1 mode softens continuously upon cooling, resembling the behaviour of this mode in undoped KT. Below 80 K, the frequency of TO1 for the doped ceramics (32 cm^−1^ @ 10 K) appears to be higher than that for KT (23 cm^−1^ @ 10 K), which is in agreement with the lower dielectric permittivity of the former if estimated according to the Lyddane–Sachs–Teller relation, $\Delta \varepsilon_{j}\Omega_{TOj}^{2}=const$ [34].

The appearance of the TO1 feature involving the mode hardening on cooling for Mn-doped KT relative to the undoped compound resembles the behaviour of this mode in ST with 2.5% Mn substituted on the Sr site [30]. In contrast, for the Mn substitution on the Ti-site, the frequency of the TO1 mode was higher than that in undoped ST over the entire temperature range. Thus, the temperature behaviour of the TO1 mode in the present ceramics is consistent with Mn residing on the cuboctahedral sites, as suggested by our XAFS analysis. At the same time, the differences between the behaviour of the TO1 mode in Mn and Li doped KT indicate that while both species reside on the K sites and exhibit significant polar displacements, the coupling of such displacements to the host lattice is weaker for Mn, resulting in dielectric relaxation with no ferroelectric transition.

## 4. Conclusions

Mn-doped KT ceramics exhibit a pronounced dielectric relaxation with the Arrhenius-law parameters *U* ≈ 105 meV and *τ*_0_ ≈ 4.6 × 10^−14^ s. This behaviour is attributed to a displacive disorder of the Mn^2+^ species occupying the oversized cuboctahedral K sites, as demonstrated using analyses of the X-ray absorption fine structure for the Mn K-edge and variable-temperature Raman spectroscopy. In the Raman spectra, the presence of polar Mn off-centring and the coupling of the resulting electric dipoles to the host lattice is manifested by the hardening of the optical, low-frequency TO1 mode at low temperatures relative to the undoped KT. A comparison with Li-doped KT, where Li^+^ cations also occupy K sites and exhibit polar displacements, indicates that the strength of such a coupling for Mn is weaker, yielding dielectric relaxation but without triggering a ferroelectric transition as observed in the Li case.

## Figures and Tables

**Table 1:** Arrhenius law parameters for the dielectric relaxation dynamics in Mn-doped KTaO_3_.

Composition	State	Arrhenius Law		Ref.
		*U* (meV)	*τ*_0_ (s)	
K_0.985_Mn_0.015_TaO_3__±δ_	ceramics	105	4.6 × 10^−14^	This work
K_0.97_Mn_0.03_TaO_3__±δ_	ceramics	120	4.2 × 10^−14^	[18]
K_1−2*x*_Mn*_x_*TaO_3_ (*x* = 0.5–5%)	ceramics	111–118	(4–11) × 10^−14^	[19]
KTaO_3_: Mn (0.01–0.1%)	single crystal	105–110	2 × 10^−14^	[14]
KTaO_3_: Mn (0.01–0.3%)	single crystal	110	2 × 10^−14^	[15]
KTaO_3_: Mn (0.01%, ESR)	single crystal	104	0.5 × 10^−14^	[15]

**Table 2:** Parameters of the Mn coordination environment in K_0.985_Mn_0.015_TaO_3±δ_ as obtained by fitting the EXAFS data (*N* coordination numbers; *R*, interatomic distances; *σ*^2^, Debye-Waller factors). In these fits, the amplitude-reduction factor was set at 1, as predicted by the FEFF calculations, and E_0_ at the energy of the 2nd pre-edge feature (Figure 2). The uncertainties (a single standard deviation) in the refined parameters, as calculated by the Artemis software, are specified in parentheses.

Valence (Site)	Shell	*N*	*R* (Å)	*σ*^2^ (Å^2^)
Mn^4+^ (Ta site)	Mn-O	6	1.9 ^a^	0.005 ^a^
	Mn-K	8	3.34(6)	0.009 ^a^
	Mn-Ta	6	3.88 ^a^	0.009 ^a^
Mn^2+^(K site)	Mn-O	3	2.34(5)	0.027 (1)
		6	2.86(7)	0.041 ^b^
		3	3.4 ^a^	0.054 ^b^
	Mn-Ta	8	3.23(2)	0.008 (2)
	Mn-K	6	3.96(2)	0.019 (5)

^a^ These parameters were kept fixed. ^b^ These values of *σ* ^2^ were defined as *σ*_2_^2^(Mn-O) = 1.5*σ*_1_^2^(Mn-O) and *σ*_3_^2^(Mn-O) = 2*σ*_1_^2^(Mn-O), where *σ*_1_, *σ*_2_, and *σ*_3_ are D-W factors for the three Mn(A)-O distances (short, medium, long) used in the model.

## Data Availability

The data presented in this study are available on request from the corresponding author.
